# Evaluation of female overactive bladder using urodynamics: relationship with female voiding dysfunction

**DOI:** 10.1590/S1677-5538.IBJU.2014.0195

**Published:** 2015

**Authors:** Kang Jun Cho, Hyo Sin Kim, Jun Sung Koh, Joon Chul Kim

**Affiliations:** 1 Department of Urology, The Catholic University of Korea, Bucheon St. Mary's Hospital, Bucheon, Korea

**Keywords:** Urinary Bladder, Overactive, Urodynamics, Urination Disorders

## Abstract

**Purpose:**

To investigate the role of urodynamic study (UDS) in female patients with overactive bladder (OAB) analyzing the relationship between OAB symptoms and female voiding dysfunction (FVD).

**Materials and Methods:**

We analyzed the clinical and urodynamic data of 163 women with OAB symptoms. OAB symptoms were categorized as dry and wet. FVD was described as detrusor underactivity (DUA), which was defined as a maximum flow rate (Q_max_) of ≤15mL/s associated with a detrusor pressure at Q_max_ (PdetQ_max_) of ≤20cmH_2_O, along with bladder outlet obstruction (BOO), which was defined as a Q_max_ of ≤15mL/s with a PdetQ_max_ of >20cmH_2_O. Clinical and urodynamic results were compared between patients with dry and wet symptoms and between those with and without FVD.

**Results:**

78 (47.9%) had dry, and 85 (52.1%) had wet symptoms. The entire group had a relatively low Q_max_ (15.1±6.6mL/s) and relatively high number of BOO (42.9%, 70/163) and DUA (8.6%, 14/163). A significantly higher number of patients with wet symptoms had detrusor overactivity compared to those with dry, as detected by the UDS (p<0.05). No significant differences were found in BOO and DUA number between dry and wet groups. Further, the international prostate symptom score did not different significantly between patients with and without FVD.

**Conclusion:**

A significant number of women with OAB had voiding dysfunction. However, the OAB symptoms themselves were not useful for predicting the presence of FVD. Therefore, UDS may be necessary for accurate diagnosis in women with OAB symptoms.

## INTRODUCTION

Overactive bladder (OAB), a symptomatic condition with urgency as the main symptom, is similar or more prevalent in women, and its overall prevalence increases with age (1, 2). Thus, with the current ageing society, concerns about OAB are rife. The initial work-up for diagnosis of lower urinary tract symptoms (LUTS) including urgency begins with examination of clinical history and frequency volume chart, physical examination, and urinalysis. In urodynamic studies (UDSs), 50% of men with LUTS show bladder outlet obstruction (BOO) due to benign prostate hyperplasia (3); therefore, the common next step for diagnosis of LUTS in men is prostatic evaluation. However, in women for whom local and systemic pathologies can be ruled out by the initial diagnostic procedure for LUTS, it is common practice to administer conservative treatment and oral pharmacotherapy for OAB without further investigation using UDS. This is because the response to antimuscarinic therapy does not differ between OAB patients with and without urodynamic diagnosis (4). UDS is usually considered when primary treatment for OAB fails, because UDS is expensive, time consuming, invasive, and sometimes inaccurate, and it is not considered to influence treatment strategy substantially (5). However, the opinions on this matter are greatly contrasting: some researchers believe that UDS is mandatory for diagnosis and treatment among women with OAB symptoms, because diagnosis based on urinary symptoms alone would lead to under-diagnosis of detrusor overactivity (DO) (6). In addition, female voiding dysfunction (FVD), which is represented by voiding and post-micturition symptoms in women, may coexist with storage symptoms (7), and pressure/ flow studies may be required for the diagnosis of this condition. In some reviews of UDS, more than 90% of women with BOO had OAB symptoms associated with obstruction (8, 9). Thus, the role of UDS in the initial diagnosis of OAB in women remains controversial.

In this study, we investigated the role of UDS in the diagnosis of women with OAB symptoms analyzing the relationship between OAB symptoms and FVD by using UDS.

## MATERIALS AND METHODS

Female patients aged ≥20 years with OAB symptoms, ≥3 points on the 5-point urinary sensation scale (10), and frequent urination (≥8 voids/24 h) who had undergone UDS were retrospectively enrolled. Those who complained of stress urinary incontinence and had diabetes mellitus, neurogenic causes, symptomatic or recurrent urinary tract infection, urinary tract calculus, history of pelvic irradiation or bladder cancer, interstitial cystitis, stage 2 or greater pelvic organ prolapse, clinically significant BOO, history of agents acting on the cholinergic or sympathetic nervous system which were given for bladder problems and prior pelvic surgery were excluded. The OAB symptoms presented were categorized as dry or wet using the 5-point urinary sensation scale. Symptoms with 3 or 4 points were OAB dry, while those with 5 points were OAB wet.

All the women underwent clinical evaluation, including complete history recording, physical examination, a 3-day bladder diary, and international prostate symptom score (IPSS), 5-point urinary sensation scale.

UDS was performed with a 6-Fr dual-lumen vesical catheter and a 12-Fr rectal balloon catheter, and the bladder was filled with 30– 50mL/min saline with the patient in the sitting position. Prior to the examination, patients were asked to void, at which point the maximum flow rate (Q_max_) in the sitting position, voiding volume, and postvoid residual urine volume (PVR) after catheterization were recorded. During bladder filling, the patients were instructed simply to report their sensations to the examiner. The presence of DO, defined as either spontaneous or provoked involuntary detrusor contractions with urgency during the filling phase of baseline cystometry (11), and total bladder capacity were recorded during filling cystometry. Detrusor pressure at the maximum flow rate (PdetQ_max_) in the voiding phase and maximum urethral closure pressure were also recorded by urethral pressure profile. Many free flows had to be abandoned because the voided volumes were too small (less than 100mL); therefore, we conducted pressure flow studies and selected the tracing with the highest Q_max_ for each patient.

FVD was described as detrusor underactivity (DUA) and BOO. DUA was defined as a Q_max_ of ≤15mL/s together with a PdetQ_max_ of ≤20cmH_2_O in the UDS. BOO was defined as a Q_max_ of ≤15mL/s with a PdetQ_max_ of >20cmH_2_O in the UDS. The clinical characteristics and UDS results were compared between the dry and wet symptom groups, and the clinical characteristics and UDS parameters were also examined in relation to the presence of FVD.

Statistical analyses were performed using SPSS 19.0 (SPSS Inc., Chicago, IL). The Student t-test and chi-square test were used where appropriate. Data are presented as means and standard deviations. A p value of <0.05 was considered statistically significant.

## RESULTS

A total of 163 female patients with OAB symptoms were enrolled in the study. Of them, 78 women (47.9%; 78/163) experienced only urgency (OAB dry), while 85 women (52.1%; 85/163) experienced OAB wet symptoms. The average age of the entire group was 59.8±13.3 years (range, 27–86 years), and the mean age was higher in the wet symptom group than in the dry one (p <0.05) (Table-1).

**Table 1:** Comparison of clinical characteristics and urodynamic parameters between patients with dry and wet OAB symptoms.

Variables	Total (n = 163)	Dry (n = 78)	Wet (n = 85)	p value
Age (year)	59.8±13.3	57.2±13.5	62.3±12.6	0.014*
Q_max_ (mL/s)	15.1±6.6	15.3±6.1	15.0±7.1	0.759
Voided volume (mL)	195.5±72.6	200.7±74.6	189.4±70.3	0.203
Residual volume (mL)	28.9±37.1	21.7±23.7	35.6±45.2	0.015*
CMG bladder capacity (mL)	232.7±104.1	250.3±92.0	216.5±112.2	0.037*
PdetQ_max_ (cm H_2_O)	31.2±13.2	31.0±10.9	31.4±15.1	0.862
MUCP (cm H_2_O)	62.1±29.3	64.4±28.1	60.1±30.5	0.346
DO (%)	63 (38.7)	23 (29.5)	40 (47.1)	0.016*
DUA (%)	14 (8.6)	5 (6.4)	9 (10.6)	0.252
BOO (%)	70 (42.9)	32 (41.0)	38 (44.7)	0.506
IPSS total	17.9±8.9	17.1±8.2	18.7±9.5	0.270
Voiding subscore	8.9±6.2	8.8±5.8	9.0±6.6	0.925
Storage subscore	9.0±3.8	8.2±3.6	9.7±3.8	0.014*
QoL score	4.5±1.2	4.1±1.2	4.9±1.1	<0.001*

**Mean±SD; Q_max_=** maximum flow rate; **PdetQ_max_ =** detrusor pressure on maximum flow; **CMG=** cystometrography; **MUCP=** maximum urethral closure pressure; **DO=** detrusor overactivity; **DUA=** detrusor underactivity; **BOO=** bladder outlet obstruction; **IPSS=** international prostate symptom score; **QoL=** quality of life,

* =statistically significant

Table-1 also shows a comparison of the clinical characteristics and urodynamic parameters between the dry and wet symptom groups. No significant differences were found in the Q_max_, voided volume, PdetQ_max_ and maximum urethral closure pressure. The PVR was higher in the wet symptom group than in the dry one, although the mean values of PVR were within the normal ranges. Maximum bladder capacity in UDS was lower in the wet symptom group than in the dry group. Although signifcant differences were found in the presence of DO via UDS, no significant differences were found in the prevalence of concurrent BOO and DUA between the groups. The storage symptom subscore and QoL score of the IPSS were higher for the wet symptom group than in the dry one.

Analysis in relation to the presence of FVD showed that the Q_max_, voided volume, and total bladder capacity were significantly lower, but PVR was significantly higher, in the FVD group than in the group without FVD. However, the total IPSS and individual subscores did not significantly differ between the groups (Table-2).

**Table 2:** Comparison of clinical characteristics and urodynamic parameters between women with OAB symptoms with and without FVD.

	No FVD (n = 77)	FVD (n = 86)	p value
Age (y)	58.2±13.1	61.3±13.3	0.130
Q_max_ (mL/s)	20.5±5.2	10.3±2.9	<0.001*
Voided volume (mL)	214.2±70.9	170.4±67.7	<0.001*
Residual volume (mL)	22.8±28.2	34.4±42.9	0.043*
CMG bladder capacity (mL)	261.9±91.1	206.4±108.4	0.001*
PdetQ_max_ (cm H_2_O)	30.2±11.2	32.0±14.7	0.377
MUCP (cm H_2_O)	62.6±30.4	61.7±28.5	0.852
IPSS total	16.7±8.3	19.0±9.2	0.101
Voiding subscore	8.0±5.8	9.6±6.4	0.110
Storage subscore	8.6±3.9	9.3±3.5	0.213
QoL score	4.3±1.2	4.6±1.1	0.164

**Mean±SD; FVD =** female voiding dysfunction; **Q_max_** = maximum flow rate; **PdetQ_max_ =** detrusor pressure on maximum flow; **CMG =** cystometrography; **MUCP =** maximum urethral closure pressure; **IPSS =** international prostate symptom score; **QoL =** quality of life;

* = statistically significant

## DISCUSSION

In this study, we found that more than half the women with OAB symptoms coexisted with voiding dysfunction by UDS, although categorizing the OAB symptoms on the basis of urgency along with urinary incontinence and IPSS was uninformative with regard to voiding dysfunction. Thus, UDS plays an important role in the diagnosis of female patients with OAB symptoms in terms of the presence of voiding dysfunction.

DO has been considered one of major characteristic of OAB, and OAB symptoms are believed to be indicative of a subsequent finding of DO on UDS. However, discordance between OAB and DO has also been reported. Digesu et al. (6) found that only 54% of women with OAB had DO on UDS, and 27% of the women with a diagnosis of DO on UDS had OAB symptoms. According to their report, because symptomatic diagnosis of OAB did not correlate with the urodynamic diagnosis of DO, symptomatic diagnosis of OAB alone was not recommendable for women with LUTS. In our study, only 38.7% (63/163) of the women with OAB symptoms had DO on UDS. When OAB symptoms were categorized by urgency urinary incontinence, DO was found significantly more frequently in the OAB wet group than in the OAB dry group (47.1% vs. 29.5%). Nevertheless, urgency incontinence was found to be suggestive of a high probability of DO among women with OAB symptoms, although DO does not affect the choice of and response to treatment among OAB patients. Nitti et al. (12) showed that the response to antimuscarinic therapy in patients with OAB symptoms was independent of the urodynamic diagnosis of DO. Therefore, we did not focus on DO in this UDS; instead, we focused on FVD.

According to our urodynamic analysis, the women with OAB symptoms, of average age 60 years, had a mean voided volume of 195.5±72.6mL and Q_max_ of 15.1±6.6mL/s. Flow rates are affected by voided volume, age, body mass index, urethral resistance, detrusor contractility, and psychological inhibition, among other factors; in particular, voided volume has a positive effect, while age has a negative effect on flow rate (13). In healthy postmenopausal continent women without DO, the Q_max_ was 23mL/s in free uroflowmetry and 18mL/s in the pressure flow study (14). Blaivas et al. (15) reported a Q_max_ value of 24.4mL/s and a voided volume of 250mL among women with an average age of 67 years who showed normal urodynamic results. Thus, the Q_max_ for the OAB patients in the present study was lower than that reported previously for healthy women. Although we did not extensively examine the factors that affect Q_max_, the relatively low voided volume due to frequent urination and voiding dysfunction might have resulted in the low Q_max_ observed in this study.

There are no generally accepted urodynamic criteria for the diagnosis of BOO in women as there are in men. Using a combined cut-off value of Q_max_ ≤15mL/s and PdetQ_max_ >20cmH_2_O for diagnosis of BOO in women, Chassagne et al. (16) reported a sensitivity of 74.3% and a specificity of 91.1% and that was the best cut-off values predicting BOO. With these criteria, the prevalence of BOO was 42.9% (70/163) in the women with OAB symptoms in our study. Although the reported prevalence of BOO in women varies widely because of the lack of standard diagnostic definitions or criteria, its prevalence has been estimated to be between 2.7% and 29% (17). Thus, the number of women with OAB symptoms who had BOO in the present study exceeded the normal range considerably. It may be affected by enrollment of women with OAB symptoms, not normal. When compared to a population of women without voiding symptoms, women with OAB might have a higher rate of BOO. Women with BOO complain of not only obstructive symptoms but also storage symptoms (18). Kayigil et al. (19) reported that BOO was more frequent in individuals with idiopathic detrusor overactivity than in the control group, although they used a different cut-off value for BOO from us. According to their study, some women with BOO reported storage symptoms as the primary complaint. In that respect, UDS can provide essential information for the differentiation of BOO from OAB.

The prevalence of DUA in women with OAB symptoms was 8.6% (14/163) in the present study. DUA is a contraction of reduced strength and/or duration, which results in prolonged bladder emptying and/or a failure to achieve complete bladder emptying within a normal time span (11). DUA may be caused by neuropathies, pelvic surgery, or drug treatments, but it is most often idiopathic. Cucchi et al. (20) reported that idiopathic DUA was found in nearly 3% adult females. Although the specific prevalence of DUA is unknown and varies widely depending on the diagnostic criteria, it seemed to be relatively high in the present study. Women with DUA usually have a high PVR and complain of obstructive symptoms (20). However, some of them have storage symptoms as the primary complaint. Without UDS, an exact diagnosis cannot be made in this situation.

The proportion of women with wet and dry OAB symptoms was almost the same in the present study. Considering that the mean age of the patients in this study was about 60 years, this result regarding the OAB symptoms is similar to that reported previously (21, 22). The mean age of the women in the wet symptom group exceeded that of the women in the dry group, which corresponds to the observation that the prevalence of wet symptoms increases significantly with advancing age (21). Chung et al. (23) evaluated urodynamic parameters in OAB subtypes and used an urgency severity scale. They reported that the mean cystometric bladder capacity was lower and PVR was higher in patients with wet symptoms than in those with dry symptoms, although the mean Q_max_ did not differ significantly between the 2 groups. The present study showed the same results. Patients with wet symptoms showed relative high PVR, although it was mostly within the normal range (less than 100mL). This means that the presence of urgency and incontinence can help predict PVR. However, the frequency of BOO or DUA did not differ significantly between the 2 groups, and FVD could not be predicted on the basis of urgency-incontinence. Therefore UDS was required to determine the presence of FVD.

Since patients with voiding dysfunction commonly complain more about voiding symptoms than storage symptoms, their IPSS voiding subscores are expected to be high. Hsiao et al. (24) reported that IPSS can be used to evaluate lower urinary tract dysfunction and can direct treatment choice for voiding dysfunction in women. Their study included women with non-stress incontinence and LUTS, but ours included only women with urgency that was rated ≥3 points on the 5-point urinary sensation scale. The total IPSS and each subscore did not differ significantly according to the existence of FVD in our study. Thus, the symptom questionnaire has limitations for application in the distinguishing of voiding dysfunction in women with OAB symptoms. Although uroflowmetry and residual volume are sometimes useful screening tools for voiding dysfunction, UDS is necessary for distinguishing the subgroups of FVD and providing the appropriate treatment. In addition, we could expect that FVD group would show worse response to the primary medical treatment for OAB, although we did not investigate the natural history or response to treatment in FVD group because of the retrospective characteristic of the study.

The present study has some limitations. The first is its retrospective nature and the lack of a control group. In practice, conducting UDSs for normal subjects is difficult, thus we could not compared the UDS of control. We set large exclusion criteria to evaluate OAB symptoms which do not related to the neurologic or anatomical factors that could be diagnosed or supposed without UDS. Thus only 163 patients could be enrolled in this study. Further, although we used a cut-off value for the UDS-based diagnosis of FVD, there is a lack of established urodynamic criteria for the diagnosis of FVD at present. In the future, prospective studies with a larger number of subjects are needed to provide more information about the role of UDS in the diagnosis of FVD in patients with OAB symptoms, with a special focus on impact upon the management of female OAB patients.

## CONCLUSIONS

A significant number of women with OAB symptoms had voiding dysfunction but only OAB symptoms are not enough to predict the presence of FVD. Therefore, UDS may be necessary for accurate diagnosis in women with OAB symptoms.

## ABBREVIATIONS

OAB =: overactive bladder
LUTS =: lower urinary tract symptoms
UDS =: urodynamic study
BOO =: bladder outlet obstruction
DO =: detrusor overactivity
FVD =: female voiding dysfunction
IPSS =: international prostate symptom score
Q_max_ =: maximum flow rate
PVR =: postvoid residual urine volume
PdetQ_max_ =: detrusor pressure at the maximum flow rate
DUA =: detrusor underactivity
